# Malaria elimination in Ghana: recommendations for reactive case detection strategy implementation in a low endemic area of Asutsuare, Ghana

**DOI:** 10.1186/s12936-023-04792-z

**Published:** 2024-01-02

**Authors:** Ebenezer Krampah Aidoo, Frank Twum Aboagye, George Edem Agginie, Felix Abekah Botchway, George Osei-Adjei, Michael Appiah, Ruth Duku Takyi, Samuel Asamoah Sakyi, Linda Amoah, George Arthur, Bernard Walter Lawson, Richard Harry Asmah, Paul Boateng, Otubea Ansah, Karen Angeliki Krogfelt

**Affiliations:** 1 Department of Medical Laboratory Technology, Accra Technical University, Accra, Ghana; 2 Bio-Medical and Public Health Research Unit, Council for Scientific and Industrial Research - Water Research Institute, Accra, Ghana; 3 Department of Molecular Medicine, Kwame Nkrumah University of Science & Technology, University Post Office, Kumasi, Ghana; 4 Department of Immunology, Noguchi Memorial Institute for Medical Research, University of Ghana, Accra, Ghana; 5 Department of Medical Laboratory, Accra Psychiatric Hospital, Accra, Ghana; 6 Department of Theoretical & Applied Biology, Kwame Nkrumah University of Science & Technology, University Post Office, Kumasi, Ghana; 7 Department of Biomedical Sciences, School of Basic and Biomedical Science, University of Health & Allied Sciences, Ho, Ghana; 8 National Malaria Elimination Programme, Accra, Ghana; 9 Department of Science and Environment, Unit of Molecular and Medical Biology, The PandemiX Center, Roskilde University, 4000 Roskilde, Denmark; 10 Department of Virus and Microbiological Special Diagnostics, Statens Serum Institut, 2300 Copenhagen, Denmark

**Keywords:** Malaria elimination, Reactive case detection, Low endemic area, Asymptomatic infections

## Abstract

**Background:**

Progress toward malaria elimination is increasing as many countries near zero indigenous malaria cases. In settings nearing elimination, interventions will be most effective at interrupting transmission when targeted at the residual foci of transmission. These foci may be missed due to asymptomatic infections. To solve this problem, the World Health Organization recommends reactive case detection (RACD). This case study was conducted to identify individuals with asymptomatic malaria, their predisposing risk factors and recommend RACD in Asutsuare, Ghana based on literature review and a cross sectional study.

**Methods:**

The study involved a search on PubMed and Google Scholar of literature published between 1st January, 2009–14th August, 2023 using the search terms “malaria” in “Asutsuare”. Furthermore, structured questionnaires were administered to one hundred individuals without symptoms of malaria and screened using rapid diagnostic test (RDT) kits, microscopy and real-time polymerase chain reaction (rt-PCR). Malaria prevalence based on the three diagnostic techniques as well as potential malaria risk factors were assessed through questionnaires in a cross-sectional study.

**Results:**

Cumulatively, sixty-four (64) studies (Google Scholar, 57 and PubMed, 7) were reviewed and 22 studies included in the literature on malaria in Asutsuare, Ghana. Significant risk factors were occupation, distance from a house to a waterbody, age group and educational level. Out of the 100 samples, 3 (3%) were positive by RDT, 6 (6%) by microscopy and 9 (9%) by rt-PCR. Ages 5–14.9 years had the highest mean malaria parasite densities of 560 parasites/µl with *Plasmodium falciparum* as the dominant species in 4 participants. Moreover, in the age group ≥ 15, 2 participants (1 each) harboured *P. falciparum* and *Plasmodium malariae* parasites. RDT had a higher sensitivity (76.54%; CI_95_ 66.82–85.54) than rt-PCR (33.33%; CI_95_ 4.33–77.72), while both rt-PCR and RDT were observed to have a higher specificity (92.55; CI_95_ 85.26–96.95) and (97.30; CI_95_ 93.87–99.13), respectively in the diagnosis of malaria.

**Conclusion:**

In Asutsuare, Ghana, a low endemic area, the elimination of malaria may require finding individuals with asymptomatic infections. Given the low prevalence of asymptomatic individuals identified in this study and as repleted in the literature review, which favours RACD, Asutsuare is a possible setting receptive for RACD implementation.

## Background

Malaria control efforts in Ghana have a long history preceding independence [1]. The global malaria elimination programme in 2016, classified Ghana as one of the nations in the control phase [2]. Subsequently, Ghana has made significant gains in reducing the disease burden [3]. Malaria parasite prevalence by microscopy in the age group 6 to 59 months decreased from 28 to 14% in 2011 and 2019, respectively [4]. In 2022, there was a further decline of 5% in the national prevalence from 14 to 9% [5]. All of these were made possible as a result of the development and implementation of numerous strategic plans of action, intervention policies, and increased financial assistance over the years [6]. Furthermore, effective 2024–2028, the National Malaria Control Programme proposes a shift in malaria control to malaria elimination with the implementation of the national malaria elimination strategic plan (NMESP) of Ghana, 2024–2028 as contained in the World Health Organization (WHO) global technical strategy (GTS) for malaria 2016–2030 [7].

In hypoendemic areas, prior to the actualization of malaria elimination, malaria programmes are confronted with the difficult task of a changing epidemiology. Such settings are characterized by an increased number of imported malaria and hence a shift in high-risk groups from young children and pregnant women to demographic and occupational risk factors [8]. Transmission also becomes more geographically focal and possibly spread by means of asymptomatic infections [9]. To deal with the problem of malaria transmission that is more demographically and geographically focal and depicted by asymptomatic infection, the WHO recommends the use of Reactive Case Detection (RACD), the screening of household members and neighbours of index cases reported in passive surveillance [10]. Studies reporting on RACD used a number of protocols to assess its effectiveness. Despite this, RACD was generally implemented in lower endemic settings [11]. According to the WHO, an annual parasite prevalence of 1–10% is considered low and ≥ 35% as high [12]. Aidoo et al. [11], in a systematic review of 55 published studies that reported on RACD from 25 countries, had no record of its implementation in Ghana. Malaria elimination is often first achieved in parts of the areas within a country and prevention of re-establishment started at the subnational level while countries are working on full interruption of transmission in all their regions [10]. For Ghana to achieve its Malaria Strategic Plan 2021–2025, of reducing malaria case incidence by 50%, malaria mortality by 90%, and achieving malaria elimination as envisaged by 2028, the first RACD strategy in Ghana, in a yet to be implemented area of Asutsuare based on a cross sectional study of preliminary findings, coupled with previous studies in the literature is proposed and recommendations made. A literature review will, therefore, synthesize experiences with malaria in Asutsuare at different time points while the cross sectional study provides additional data to collectively inform recommendations and future use of RACD in Asutsuare, Ghana. This case study aimed to identify individuals with asymptomatic malaria, their predisposing risk factors and recommendations for the feasibility of RACD in a low-endemic area of Asutsuare in future. Within this context, understanding the epidemiology of low-endemic malaria transmission is crucial to achieving and sustaining elimination.

## Ghana and Asutsuare’s Malaria landscape

In Ghana, malaria is endemic and perennial. Children under five years and pregnant women are the most vulnerable. Malaria is marked by seasonal fluctuations which differ from region to region [4]. In the ecological zone of Northern Savannah area, parasite prevalence is highly seasonal and peaks in a single wet season (June–October). However, in both forest and coastal ecological areas, malaria parasite prevalence peaks twice in a year [13]. Members of the *Anopheles gambiae* species complex, such as *An. gambiae, Anopheles coluzzii*, and *Anopheles arabiensis*, are the main malaria vectors in Ghana. *Anopheles gambiae* predominate and transcends across the country. However, during the drier months at the start and end of the season, *An. coluzzii* is frequently more prevalent. *Anopheles funestus* is a minor vector present at low levels. The vector species tend to bite late at night and are more prevalent in rural and periurban locations. The majority of parasite species, more than 95.0%, are *Plasmodium falciparum*. Although *Plasmodium vivax* infections have not yet been recorded, low-level *Plasmodium malariae* and *Plasmodium ovale* infections have been observed, typically occurring as mixed infections with *P. falciparum* [4].

Asutsuare is in the Shai-Osudoku District, Greater Accra Region, Ghana. It has a low seasonal malaria transmission, which peaks slightly after the rainy season between April to July [14]. Parasite prevalence by light microscopy was 2.4% in February 2009, 2.7% in May 2009 and 2.4% in August 2009 during a third survey [15]. Also in 2009, microscopy prevalence was reported to be 8.9% [16]. Regarding age groups, parasite prevalence increased from 1.7% in < 5 years to 3.4% in the 5–14 year group and 0% in the adult group by microscopy [14]. Another study reported a microscopy prevalence of 3.5% [17]. Agbana et al. [18] recorded a parasite prevalence of 0%, 2.2%, 5.6%, 11.2% and 50.1% by RDT, microscopy, PET-PCR, HRP2 bead assay, nested PCR, respectively.

A survey in the Asutsuare community comprising 337 respondents also indicated universal awareness of malaria as a health problem coupled with high knowledge of some common malaria symptoms. However, 3% of such respondents visited a health facility with suspected malaria and the remainder (97%) visited following self-medicated treatment failure [19]. Lamptey et al. [20] reported a 96% parasite distribution of *P. falciparum* and less than 5% *P. malariae* and *P. ovale* single infection and mixed infections of *P. falciparum* and *P. malariae/P. ovale*. Comparatively, *P. falciparum* prevalence was 6.4%, 8.0% and 16.4% amongst adults, children and pregnant women respectively. While children recorded a gametocyte prevalence of 39.5% (15/38), adult and pregnant women had a gametocyte prevalence of 17.4% (4/23) and 29.7% (11/37) respectively. Following a baseline parasite prevalence of [2.9%, (6/209)], *P. falciparum* proportion of children with at least one episode of malaria at the end of a follow-up period was 8.6% (95% CI 5.5–13.3) [21]. In a study involving 57 children, 18 had asymptomatic *P. falciparum* infections, 22 with symptomatic malaria, and 17 devoid of *P. falciparum* by microscopy or rapid diagnostic test [22]. In another follow-up study, children with malaria (168/669; 25.1%) were identified as either febrile (101/669; 15.1%) or those with asymptomatic parasitaemia (67/669; 10.0%) [23]. In children with *P. falciparum* malaria (n = 25), malaria was diagnosed by microscopy as either febrile (> 37.5 °C) uncomplicated *P. falciparum* infection (n = 7) or asymptomatic parasitaemia (n = 18) [24].

Walker et al. [25], in a study involving 78 healthy adults (18–69 years) likely to have been exposed many times to *P. falciparum*, found none of them to be positive by rapid diagnostic test (RDT) and microscopy. Furthermore, in two longitudinal cohort surveys, the parasite prevalence was found to be 14.45% [26] and 13% (53/395 children) cumulative incidence in a prospective cohort study [27]. Overall asexual parasite prevalence by microscopy of 6.1% (11/180) and gametocyte prevalence of 0% (0/180) were recorded by Jones et al. [28]. Ravens et al. [29] in their study reported a malaria prevalence of 8.9%. In a study involving 115 participants, *P. falciparum* microscopy prevalence was found to be 17.2% at any point during the respective study periods [26]. Tiendrebeogo et al. [30] recorded a malaria incidence of 15.1%. Relative to younger children (1–5 year old), older children (6–12 year old) were protected from febrile malaria with no association in parasitaemia (P = 0.29). Moreover, using 18srRNA PCR for *P. falciparum* speciation, 59.1% of samples tested positive for malaria parasite with a microscopy prevalence of 3.3% and RDT of 1.7% [31].

Abukari et al. [32] also reported asymptomatic *P. falciparum* microscopy prevalence of 3.75% (3/80). During the dry and rainy seasons, Acquah et al. [33] using Pfs25 mRNA PCR reported a gametocyte prevalence of 3/230 (1.3) and 3/174 (1.7), respectively. Conversely, the same study had a microscopy asexual parasite prevalence of 8/230 (3.5) and 7/174 (4.0) respectively. Furthermore, out of 644 pregnant women evaluated for placental malaria, peripheral parasitaemia was detected in 104 of them [34]. Ononye et al. [35] observed higher proportion of *An. gambiae* biting indoors relative to outdoors, in addition to sporozoite rate of 0.24% and an overall annual Entomological Inoculation Rate (EIR) of 4.9 infective bites per man per year (ib/m/y).

Generally, as indicated in the literature, malaria is a problem in Asutsuare despite its low endemicity. Hence, the need to recommend RACD that is informed by 14 years of literature between 2009 and 2023 in the country’s efforts towards malaria elimination.

### Study design

Between July, 2022 to August, 2022, a cross sectional community-based study of asymptomatic individuals of all age groups was conducted in Asutsuare. This was further complemented with a search on PubMed and Google Scholar of literature published between 1st January, 2009–14th August, 2023 (effective year for the synthesized experiences with malaria in Asutsuare to the end of the literature search that met the inclusion criteria detailed below) using the search terms “malaria” in “Asutsuare”.

Search results were assessed using the following inclusion criteria:

1. Studies on the immunology, entomology, ecology, or genetics of malaria.
2. Studies designed to determine prevalence based on the diagnostic testing method used or characterize transmission patterns in all age groups.
3. Studies other than malaria were excluded.

### Sample size determination

The sample size with a 95% CI and 5% margin of error was estimated according to the formula and variables defined, **N** =  **Z**^**2**^**pq/e**^**2**^, **Z** − 1.96, assuming 6% malaria prevalence from previous study [18] and 15% expected refusal rate. Following these assumptions, the final sample size was determined to be 100.

### Data collection and laboratory methods

Data collected on the structured questionnaire included whether the household has been sprayed with insecticide, bed net ownership and usage, gender, age, amongst others. A single finger prick was used to collect blood samples, which were then spotted on Whatman 903™ protein saver card for rt-PCR and processed into smears for both thin and thick films. Using standard procedures, asexual parasites in thick and thin blood films on the same slide were counted against a minimum of 200 white blood cells and multiplied by a white blood cell count of 8000 per microlitre for quantification of parasite density [36]. For rt-PCR, dried blood spots on Whatman 903™ protein saver cards were placed inside individually sealed zip locks containing desiccant and kept at − 20 °C. As previously described [37], DNA was extracted using the Chelex method and kept at − 20 °C. *P. falciparum* rt-PCR was performed per published protocols [38].

### Statistical analyses

Data collected was entered into Microsoft Excel 2019 (Microsoft Corp., Washington, USA) and analysed using Statistical Package for the Social Sciences (SPSS) version 26 (IBM Corp., Armonk, NY, USA) and GraphPad Prism 9.0 (GraphPad Software Inc., Boston, USA). For categorical variables, frequencies and percentages were computed while descriptive statistics was done for continuous variables. Test of association between variables was done using t-test, chi-square and analysis of variance where appropriate. Risk factors associated with malaria infection amongst the cohort was explored using multivariate analysis. A p-value < 0.05 was considered statistically significant. MedCalc® Statistical Software version 22 (MedCalc Software Ltd, Ostend, Belgium) was used to evaluate the diagnostic performance of rt-PCR and RDT for the detection of *Plasmodium* species infection. Positive and negative predictive values of rt-PCR and RDT were computed taking into consideration the prevalence of disease. The level of agreement between the diagnostic tests and the gold standard was measured using the Cohen Kappa score as described by McHugh [39].Values between 0.0 and 0.20 were considered as poor agreement, 0.21–0.40 as fair, 0.41–0.60 as moderate, 0.61–0.80 as strong and 0.81 and 1.00 as nearly perfect.

### Ethics

**Fig. 1:**
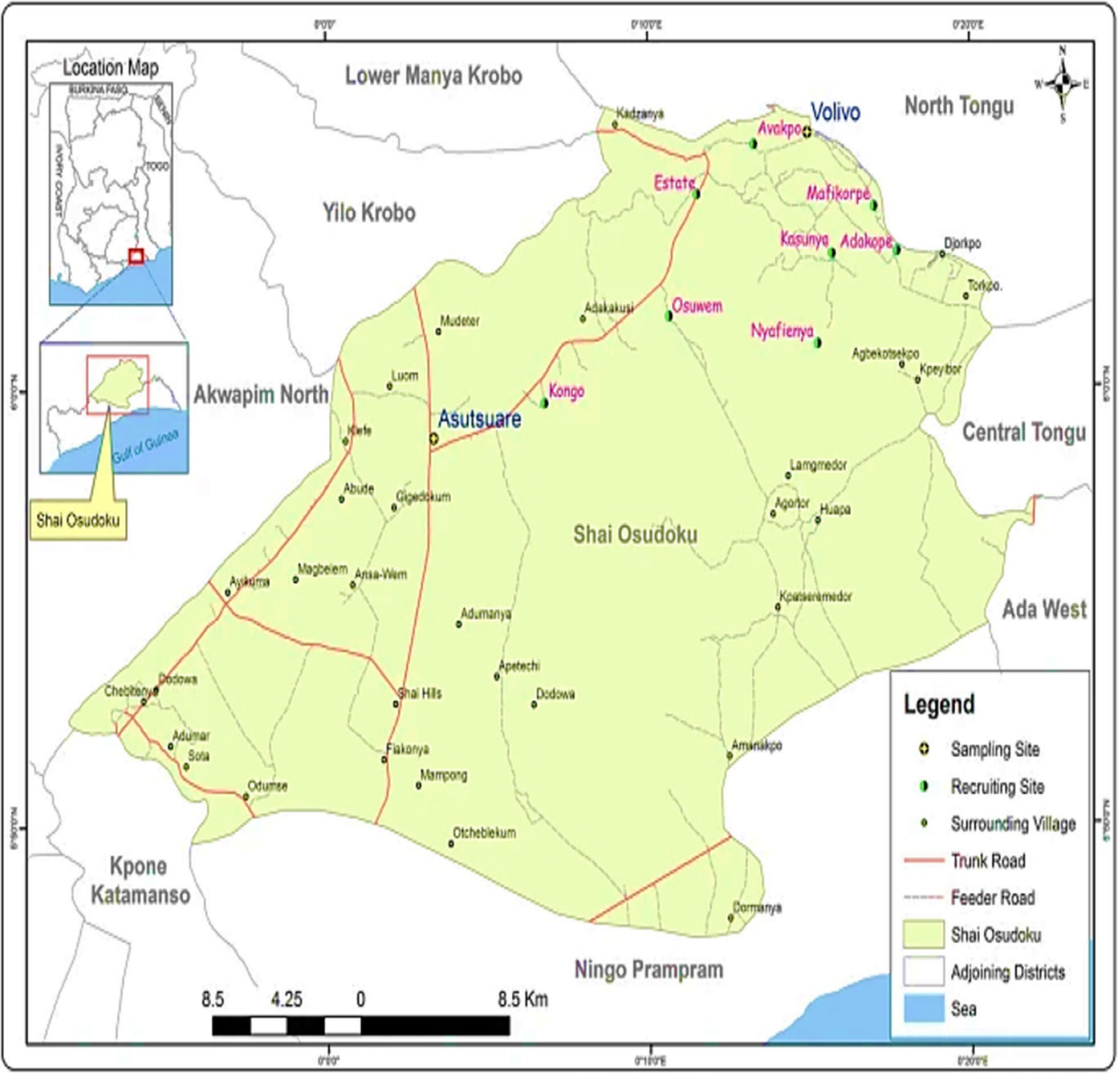
Map of Asutsuare in the Shai Osudoku District, Greater Accra Region, Ghana “adapted with permission from [ 20 ].

The study was approved by the Research and Ethical Review Committee of the Department of Medical Laboratory Technology, Accra Technical University (ATU/MLT/ET/01192135B/2021–2022). Informed consent was expressly sought from all adult participants. Also, participants below 18 years old were given an assent form to be completed by their parents or guardians on their behalf. During screening, participants who tested positive for malaria were sent to the Shai Osodoku Health Centre and treated according to current national malaria treatment guidelines (Fig. 1).

## Results

### Demographic characteristics of study participants

The study recruited a total of 100 participants who were asymptomatic of malaria. As shown in Table 1, many of the study participants were females, representing 55%. Study participants > 15 years were 53% representing the majority of the study population, while ages 5–14.9 years and ≥ 15years were 37% and 10% respectively. More than half, 52%, of participants were without bednets while 53 (53%) lived near water bodies.

**Table 1:** Characteristics of the study population

Characteristics	Category	N	%
Gender	Male	45	45.0
	Female	55	55.0
Age groups (years)	0–4.9	10	10.0
	5–14.9	37	37.0
	> 15	53	53.0
Educational level	None	17	17.0
	Primary	76	76.0
	Secondary	7	7.0
Occupation	Unemployed	94	94.0
	Forest related	6	6.0
Work place distance to forest	None	92	92.0
	< 1 km	8	8.0
Live near water body	No	47	47.0
	Yes	53	53.0
Owns a bed net	Yes	48	48.0
	No	52	52.0

### Prevalence of *Plasmodium* infection and distribution of parasite density

**Fig. 2:**
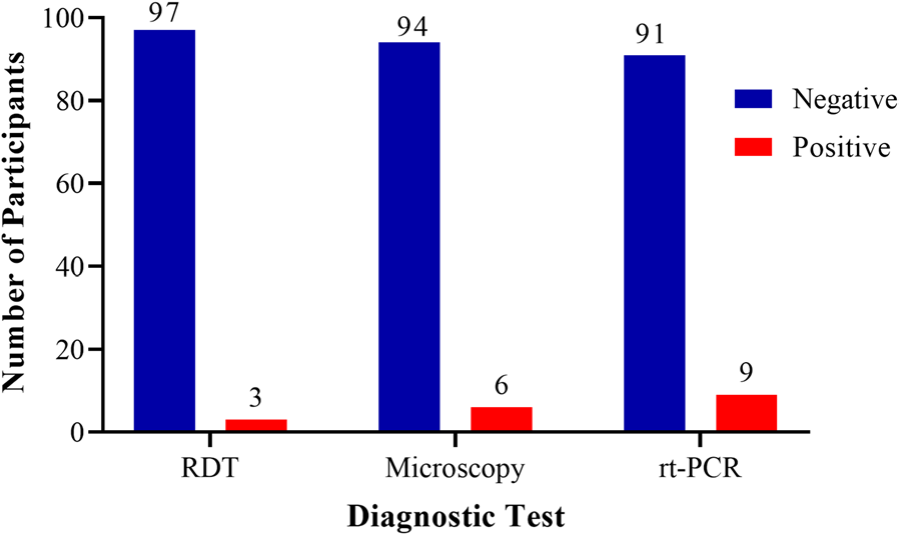
Distribution of Plasmodium infection by diagnostic tests

**Fig. 3:**
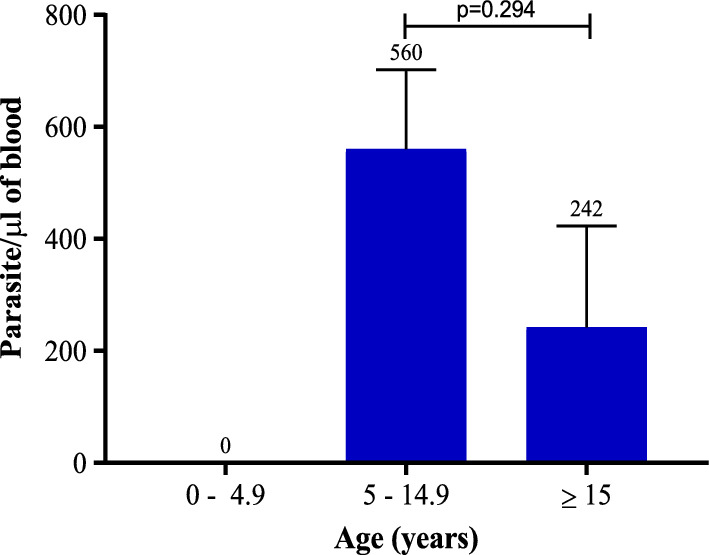
Distribution of parasite density amongst study participants by age

*Plasmodium* infection using three diagnostic techniques showed a prevalence of 3%, 6% and 9% by RDT, microscopy and rt-PCR, respectively (Fig. 2). The study found no *Plasmodium* infection in participants aged 0–4.9 years. However, the mean parasite density for participants aged 5-14.9 years was 560 parasite/µl of blood and participants ≥ 15 years had a mean parasite density of 242 parasite/µl of blood. Statistically, there was no significant difference (p = 0.294) in parasite density between participants aged 5-14.9 years and those ≥ 15 years (Fig. 3).

### Distribution of *Plasmodium* infection amongst study participants using microscopy and rt-PCR

Regarding the 6 participants who had malaria, all 6 had a forest related job such as farming and all 6 had their houses not sprayed. Four did not own a bednet and all 6 travelled 2 weeks prior to the survey with a household member. Using microscopy, statistically occupation and distance of house to a water body were found to be significant (< 0.001 and 0.043), respectively (Table 2).

**Table 2:** Distribution of *Plasmodium* infection amongst study participants using microscopy

Characteristics	Category	Microscopy	
		n (%)	χ^2^(p-value)
Gender	Male	4 (8.9)	
	Female	2 (3.6)	1.211 (0.271)
Age groups (years)	0–4.9	0 (0.0)	2.622 (0.269)
	5–14.9	4 (66.6)	
	≥ 15	2 (33.4)	
Educational level	None	1 (5.9)	0.492 (0.782)
	Primary	5 (6.6)	
	Secondary	0 (0.0)	
Occupation	Unemployed	0 (0.0)	100 (< 0.001)
	Forest related	6 (100.0)	
Workplace distance to forest	None	6 (6.5)	0.555 (0.486)
	< 1 km	0 (0.0)	
Live near water body	No	3 (6.4)	0.023 (0.879)
	Yes	3 (5.7)	
Owns a bed net	Yes	2 (4.2)	0.059 (0.808)
	No	4 (7.7)	
Sleepover at work	Yes	–	NA
	No	6 (100.0)	
Distance of house to a water body	None	3 (50.0)	6.271 (0.043)
	< 100 m	1 (16.7)	
	100–499 m	2 (33.3)	
House distance to forest	None	5 (83.3)	0.188 (0.664)
	< 1 km	1 (16.7)	
Travel within 2 weeks	Yes	6 (100.0)	0.064 (0.800)
	No	0 (0.0)	
Travel with a household member	Yes	6 (100.0)	0.064 (0.800)
	No	0 (0.0)	
Sleep outside the previous night	Yes	-	NA
	No	6 (100.0)	
House sprayed	Yes	-	NA
	No	6 (100.0)	
Source of drinking water	River	0 (0.0)	0.789 (0.674)
	Borehole	0 (0.0)	
	Pipe	6 (100.0)	
Other mosquito bite prevention methods	Nothing	0 (0.0)	0.954 (0.917)
	Fan	0 (0.0)	
	Use of coil	6 (100.0)	
	Coil/fan/screened Window	0 (0.0)	
	Use coil/spray	0 (0.0)	

*
NA* chi-square test could not be computed

Out of the 9 participants, identified by rt-PCR to harbour malaria parasites, 8 were within the age group 5–14.9 and 1 was ≥ 15 years. Five did not own a bednet and lived near a waterbody. Age group and education were found to be statistically significant (0.003 and 0.032), respectively (Table 3).

**Table 3:** Distribution of *Plasmodium* infection amongst study participants using rt-PCR

Characteristics	Category	rt-PCR	
		n (%)	χ^2^ (p-value)
Gender	Male	6 (66.7)	1.876 (0.171)
	Female	3 (33.3)	
Age groups (years)	0–4.9	0 (0.0)	11.460 (0.003)
	5–14.9	8 (88.9)	
	≥ 15	1 (11.1)	
Educational level	None	1 (11.1)	4.615 (0.032)
	Primary	8 (88.9)	
	Secondary	0 (0.0)	
Occupation	Unemployed	7 (77.8)	1.110 (0.574)
	Forest related	2 (22.2)	
Work place distance to forest	None	8 (88.9)	0.130 (0.718)
	< 1 km	1 (11.1)	
Live near water body	No	4 (44.4)	0.260 (0.872)
	Yes	5 (55.6)	
Owns a bed net	Yes	4 (44.4)	0.050 (0.823)
	No	5 (55.6)	
Sleepover at work	Yes	–	NA
	No	9 (100.0)	
Distance of house to a water body	None	4 (44.4)	0.134 (0.935)
	< 100 m	4 (44.4)	
	100–499 m	1 (11.2)	
House distance to forest	None	6 (66.7)	0.472 (0.492)
	< 1 km	3 (33.3)	
Travel within 2 weeks	Yes	0 (0.0)	0.100 (0.752)
	No	9 (100.0)	
Travel with a household member	Yes	0 (0.0)	0.100 (0.752)
	No	9 (100.0)	

*
NA* chi-square test could not be computed

### Distribution of *Plasmodium* spp infection amongst study participants

As shown in Table 4, P. *falciparum* was the predominant species identified, followed by *P. malariae*. The study, however, did not find *P. ovale* and *P. vivax.* The highest prevalence of *P. falciparum* was seen in males 3(60%), primary students 4(80%), age group 5-14.9 years 4 (80%), forest related jobs 5(100%), workplace distance not near forest 5(100%), those who do not live near water body 3(60%) and do not own a bed net 3(60%).

**Table 4:** Distribution of *Plasmodium* spp infection amongst study participants

Characteristics	Category	Microscopy				
		Mps	Pf	Po	Pm	Pv
		n (%)	n (%)	n (%)	n (%)	n (%)
Gender	Male	4 (66.7)	3 (60.0)	0 (0.0)	1(100.0)	0 (0.0)
	Female	2 (33.3)	2 (40.0)	0 (0.0)	0 (0.0)	0 (0.0)
Age groups (years)	0–4.9	0 (0.0)	0 (0.0)	0 (0.0)	0 (0.0)	0 (0.0)
	5–14.9	4 (66.7)	4 (80.0)	0 (0.0)	0 (0.0)	0 (0.0)
	≥ 15	2 (33.3)	1 (20.0)	0 (0.0)	1(100.0)	0 (0.0)
Educational level	None	1 (16.7)	1 (20.0)	0 (0.0)	0 (0.0)	0 (0.0)
	Primary	5 (83.3)	4 (80.0)	0 (0.0)	1(100.0)	0 (0.0)
	Secondary	0 (0.0)	0 (0.0)	0 (0.0)	0 (0.0)	0 (0.0)
Occupation	Unemployed	0 (0.0)	0 (0.0)	0 (0.0)	0 (0.0)	0 (0.0)
	Forest related	6 (100.0)	5 (100.0)	0 (0.0)	1(100.0)	0 (0.0)
Work place distance to forest	None	6 (100.0)	5 (100.0)	0 (0.0)	1(100.0)	0 (0.0)
	< 1 km	0 (0.0)	0 (0.0)	0 (0.0)	0 (0.0)	0 (0.0)
Live near water body	No	3 (50.0)	3 (60.0)	0 (0.0)	0 (0.0)	0 (0.0)
	Yes	3 (50.0)	2 (40.0)	0 (0.0)	1(100.0)	0 (0.0)
Owns a bed net	Yes	2 (33.3)	2 (40.0)	0 (0.0)	0 (0.0)	0 (0.0)
	No	4 (66.7)	3 (60.0)	0 (0.0)	1(100.0)	0 (0.0)

### Clinical performance of rt-PCR and RDT for Malaria diagnosis

In Table 5, both rt-PCR and RDT showed higher specificity (92.55; CI_95_ 85.26–96.95) and (97.30; CI_95_: 93.87–99.13) respectively. Also, rt-PCR had a higher negative predictive value (93.35% CI_95_ 88.83–96.12) while RDT showed a positive predictive value (92.53% CI_95_ 83.80-96.74). An accuracy of 91.05% (CI_95_ 86.61–93.43) was reported for RDT and 87.22% (CI_95_ 79.06–93.06) was reported as the accuracy of rt-PCR in diagnosing malaria parasites infection.

**Table 5:** Clinical performance of rt-PCR and RDT in the diagnosis of malaria using microscopy as the gold standard

Clinical Performance Indicators		Diagnostic techniques	
		rt-PCR	RDT
Sensitivity	%	33.33	76.54
	CI_95_	4.33–77.72	66.82–85.54
Specificity	%	92.55	97.30
	CI_95_	85.26–96.95	93.87–99.13
Positive Predictive Value	%	30.69	92.53
	CI_95_	10.41–62.77	83.80–96.74
Negative Predictive Value	%	93.35	90.56
	CI_95_	88.83–96.12	86.61–93.43
Accuracy	%	87.22	91.05
	CI_95_	79.06–93.06	86.97–94.18
ROC Analysis	AUC	0.67	0.57
	CI_95_	0.36–0.89	0.31–0.83
	Standard error	0.13	0.029
	p-value	0.344	0.589
Cohen’s Kappa	Κ	0.21	0.19
	Standard error	0.16	0.18
	p-value	0.032	0.054

CI_95_, 95% Confidence Interval; ROC, Receiver Operator Characteristics; AUC , Area Under Curve

## Discussion

The study sought to identify asymptomatic malaria and the predisposing risk factors in a low-endemic area of Asutsuare using microscopy, RDT, and rt-PCR. Primarily, malaria risk factors were occupation, distance of a house to a water body, age group and educational level. All six individuals who tested positive by microscopy had forest-related occupation and hence possible exposure to mosquitoes in the course of work. This finding is consistent with other studies in which adult men in low-transmission settings were at risk due to forest-related occupation and behavioural factors [8, 9, 40]. In Swaziland, distance of a house to a water body was one of the most important variables for prediction of transmission risk [41].

Prevalence as found by RDT, microscopy and rt-PCR was 3%, 6% and 9% respectively. In a study at Asutsuare, Agbana et al. [18] reported a parasite prevalence of 0%, 2.2% and 5.6% by RDT, microscopy and PET-PCR, respectively. The differences in the prevalence results may possibly be because of the period both studies took place. Be that as it may, both studies pointed to a low malaria prevalence. According to Ajakaye and Ibukunoluwa [42], a high prevalence leads to a high positive predictive value and low negative predictive value. However, in the present study, using microscopy as the gold standard, RDT showed a low prevalence, high positive predictive value (92.53%) and high negative predictive value (90.56%).

Also, RDT had a sensitivity and specificity of 76.54% and 97.30% respectively. The high positive predictive value demonstrates the utility of RDT in resource limited settings for the diagnosis of malaria, with positive results able to be considered truly positive. Also, the high negative predictive value minimizes false negatives. The WHO recommends 95% sensitivity and 97% specificity for malaria RDT. This is worth considering when utilizing RDT for malaria control programmes or for the diagnosis of malaria. In this study, the RDT specificity of 97.30% fell within the recommendation of the WHO, while that of the 76.54% sensitivity fell short of the recommendation. Compared with the WHO recommendation, the low sensitivity could be as a result of low parasite density below the threshold of RDT positivity [43]. The prozone effect, hyperparasitaemia, deletion or mutation of the HRP-2 gene, and other factors have also been cited as possible causes of low sensitivity in other studies [44, 45].

In hypoendemic areas, a negative RDT result therefore calls for confirmation with more sensitive diagnostic techniques as demonstrated by the study’s low sensitivity. The high specificity in this study suggests that community health workers in primary healthcare centers may employ RDT to rule out the absence of malaria in situations where microscopes are uncommon or lacked trained microscopist. The use of PCR in the diagnosis of malaria has been explored in many studies and indicated as a sensitive tool [46, 47]. This study detected a rt-PCR sensitivity and specificity of 33.3% and 92.55%, respectively, when using microscopy as gold standard. In contrast to this study, Madkhali et al. [48] reported a higher PCR sensitivity and specificity of 97.6% and 95.5% respectively, than what was reported in this study. This variation could be attributed to differences in study area, sample size and the type of nucleic acid isolation and amplification kit used.

Asymptomatic *Plasmodium* infection was prevalent in males than in females. Similar to this study, Mensah et al. [49] found that males were more likely to have malaria than female. Males typically have a weaker immunological response than females [50] and this may have necessitated this observation [51]. Such immunological differences may find expression in oestradiol, testosterone and progesterone [50]. In the host, however, hormonal concentrations may also be generated and modified by parasites [51]. Moreover, males are more likely to stay late outside at night and at best sleep outside without a mosquito net or proper protective clothing. Therefore, a better understanding of how gender activities affect exposure to mosquitoes can help inform guidelines for reducing malaria infection, particularly in males.

Asymptomatic malaria carriage has been associated with age [52]. Age-wise, majority of the participants with *Plasmodium* infection in this study were between 5 and 14.9 years. Studies conducted in Northern Ghana by Kanwugu et al. [52], was not consistent with the findings of the present study. However, the findings of Aidoo et al. [53] was consistent with the present study. Age is regarded as one of the most significant factors influencing protective immunity. Comparatively, younger children are most susceptible, while young children and adults who have had frequent bouts of malaria and built up immunity are more likely to carry asymptomatic infection [54].

### Recommendations for consideration of RACD

At the onset of RACD, awareness should be raised to achieve high coverage and to encourage community participation within the defined target area. It will be ideal to have community health workers at the forefront of this campaign to have the buy in of the community. Such an awareness drive should include malaria symptoms, preventive measures, and the importance of seeking early diagnosis and treatment.

There is the need for a dedicated research team comprising healthcare providers stationed at the recruiting medical facility, laboratories, and community health workers for swift notification and investigation of index cases. These personnels should have the capacity to conduct case investigations, perform rapid diagnostic tests (RDTs), and administer appropriate treatment based on national treatment guidelines. Furthermore, there should be active collaboration among these personnels at the various levels (primary healthcare centres and hospitals) and other stakeholders to coordinate efforts and leverage available resources. Partnerships can enhance the scope and impact of RACD.

There should be clear definition of the target area. Hence, in resource limited settings, RACD should be restricted to particular high-risk places, such as within known foci or in places with low coverage of indoor residual spraying (IRS) and/or insecticide-treated nets (ITN). In like manner, index case households and close neighbours of index cases should be given precedence. Where malaria risk may not be connected to place of residence but rather population characteristics such as occupation, RACD can be implemented demographically instead of geographically, reactively testing contacts of members with common risk factors.

During RACD, to optimize coverage, screening should be undertaken when there is a full house or majority of the household members are likely to be present (such as during vacation or days people do not go to the farm). Also, those absent should be noted and possibly followed up on and screened later to avoid missing those at highest risk of contracting malaria.

RACD should be enhanced by frequently monitoring and evaluating its effectiveness by treatment outcomes, the number of cases detected and challenges faced during implementation.

## Conclusion

For targeted interventions, risk factor assessments in low-endemic areas with a goal of eliminating malaria are crucial. Routine data analysis as depicted by this study can be used to identify risk factors and further enhanced with RACD as recommended by the study in malaria investigations.

## Data Availability

The datasets used and/or analysed during the current study are available from the corresponding author on reasonable request.

## Abbreviations

EIR: Entomological inoculation rate
GTS: Global technical strategy
HRP_2_: Histidine rich protein 2
Mps: Malaria parasites
mRNA: Messenger ribonucleic acid
NMESP: National malaria elimination strategic plan
PET-PCR: Photo-induced electron transfer polymerase chain reaction
RACD: Reactive case detection
RDT: Rapid diagnostic test
rt-PCR: Real time polymerase chain reaction
SPSS: Statistical Package for the Social Sciences
WHO: World Health Organization
